# Perceived Cognitive Deficits in Patients With Symptomatic SARS-CoV-2 and Their Association With Post–COVID-19 Condition

**DOI:** 10.1001/jamanetworkopen.2023.11974

**Published:** 2023-05-01

**Authors:** Teresa C. Liu, Sun M. Yoo, Myung S. Sim, Yash Motwani, Nisha Viswanathan, Neil S. Wenger

**Affiliations:** Division of General Internal Medicine & Health Services Research, Department of Medicine, University of California, Los Angeles; Division of General Internal Medicine & Health Services Research, Department of Medicine, University of California, Los Angeles; Department of Medicine Statistics Core, University of California, Los Angeles; Department of Medicine Statistics Core, University of California, Los Angeles; Division of General Internal Medicine & Health Services Research, Department of Medicine, University of California, Los Angeles; Division of General Internal Medicine & Health Services Research, Department of Medicine, University of California, Los Angeles

## Abstract

**IMPORTANCE:**

Neuropsychiatric symptoms are common in acute SARS-CoV-2 infection and in post–COVID-19 condition (PCC; colloquially known as long COVID), but the association between early presenting neuropsychiatric symptoms and PCC is unknown.

**OBJECTIVE:**

To describe the characteristics of patients with perceived cognitive deficits within the first 4 weeks of SARS-CoV-2 infection and the association of those deficits with PCC symptoms.

**DESIGN, SETTING, AND PARTICIPANTS:**

This prospective cohort study was conducted from April 2020 to February 2021, with follow-up of 60 to 90 days. The cohort consisted of adults enrolled in the University of California, Los Angeles, SARS-CoV-2 Ambulatory Program who had a laboratory-confirmed symptomatic SARS-CoV-2 infection and were either hospitalized in a University of California, Los Angeles, hospital or one of 20 local health care facilities, or were outpatients referred by a primary care clinician. Data analysis was performed from March 2022 to February 2023.

**EXPOSURE:**

Laboratory-confirmed SARS-CoV-2 infection.

**MAIN OUTCOMES AND MEASURES:**

Patients responded to surveys that included questions about perceived cognitive deficits modified from the Perceived Deficits Questionnaire, Fifth Edition, (ie, trouble being organized, trouble concentrating, and forgetfulness) and symptoms of PCC at 30, 60, and 90 days after hospital discharge or initial laboratory-confirmed infection of SARS-CoV-2. Perceived cognitive deficits were scored on a scale from 0 to 4. Development of PCC was determined by patient self-report of persistent symptoms 60 or 90 days after initial SARS-CoV-2 infection or hospital discharge.

**RESULTS:**

Of 1296 patients enrolled in the program, 766 (59.1%) (mean [SD] age, 60.0 [16.7] years; 399 men [52.1%]; 317 Hispanic/Latinx patients [41.4%]) completed the perceived cognitive deficit items at 30 days after hospital discharge or outpatient diagnosis. Of the 766 patients, 276 (36.1%) perceived a cognitive deficit, with 164 (21.4%) having a mean score of greater than 0 to 1.5 and 112 patients (14.6 %) having a mean score greater than 1.5. Prior cognitive difficulties (odds ratio [OR], 1.46; 95% CI, 1.16–1.83) and diagnosis of depressive disorder (OR, 1.51; 95% CI, 1.23–1.86) were associated with report of a perceived cognitive deficit. Patients reporting perceived cognitive deficits in the first 4 weeks of SARS-CoV-2 infection were more likely to report symptoms of PCC than those without perceived cognitive deficits (118 of 276 patients [42.8%] vs 105 of 490 patients [21.4%]; χ_1_^2^, 38.9; *P* < .001). Adjusting for demographic and clinical factors, perceived cognitive deficits in the first 4 weeks of SARS-CoV-2 were associated with PCC symptoms (patients with a cognitive deficit score of >0 to 1.5: OR, 2.42; 95% CI, 1.62–3.60; patients with cognitive deficit score >1.5: OR, 2.97; 95% CI, 1.86–4.75) compared to patients who reported no perceived cognitive deficits.

**CONCLUSIONS AND RELEVANCE:**

These findings suggest that patient-reported perceived cognitive deficits in the first 4 weeks of SARS-CoV-2 infection are associated with PCC symptoms and that there may be an affective component to PCC in some patients. The underlying reasons for PCC merit additional exploration.

## Introduction

The world continues to grapple with the diverse clinical manifestations of SARS-CoV-2 infection and patients who have persistent symptoms. A key question is why some patients with SARS-CoV-2 have persistent symptoms, which the Centers for Disease Control and Prevention defines as symptoms that extend beyond 4 weeks after the initial infection.^1^ Post–COVID-19 condition (PCC; colloquially known as long COVID) is characterized by many symptoms, of which cognitive impairment is a frequent complaint.^2–8^ So-called brain fog in particular has been a common and debilitating symptom^9^ affecting all age groups. Other neurocognitive symptoms associated with PCC include memory problems, difficulty concentrating, trouble focusing, and posttraumatic stress disorder.^10–13^ Furthermore, anxiety and depression are commonly reported alongside PCC.^14–16^

Most studies evaluating cognitive dysfunction in patients with SARS-CoV-2 have focused on the clinical characterization of cognitive symptoms associated with acute SARS-CoV-2 and PCC.^4,5,9,17–19^ With respect to both the acute and postacute sequelae of SARS-CoV-2, few studies have examined potential factors associated with the development of cognitive dysfunction. Given the potential long-term impact of neurocognitive deficits on quality of life and productivity,^5^ it is important to understand potential factors associated with cognitive dysfunction during the acute phase of SARS-CoV-2. Furthermore, some retrospective studies^20–22^ and a 2022 prospective analysis^23^ demonstrate that premorbid distress is associated with PCC symptoms at 4 weeks after initial infection or later, yet the association between early development of cognitive symptoms and the development of PCC is not understood. In this cohort study, we evaluated data from a clinical cohort of patients with SARS-CoV-2 who were surveyed concerning perceived cognitive deficits.^24^ We explored the perceived level of cognitive deficit, factors associated with those deficits in the acute phase of SARS-CoV-2 infection, and the association of those deficits with the development of PCC.

## Methods

This study was approved by the University of California, Los Angeles (UCLA) institutional review board with no requirement of informed consent due to the nature of the study as a retrospective analysis of deidentified clinical data in accordance with 45 CFR § 46. This study also follows the Strengthening the Reporting of Observational Studies in Epidemiology (STROBE) reporting guideline.^25^The UCLA SARS-CoV-2 Ambulatory Program enrolled a longitudinal, prospective cohort of adults with laboratory-confirmed SARS-CoV-2 infection from April 2020 to February 2021.^24^ Patients were followed up for clinical purposes with standardized questionnaires administered by nurses via telephone at 30, 60, and 90 days after hospital discharge or from the date of a positive SARS-CoV-2 test for nonhospitalized patients. Patients hospitalized for SARS-CoV-2 were discharged from UCLA hospitals or 20 other local health care facilities. Ambulatory patients were referred by a primary care clinician. A multidisciplinary team followed this cohort to address persistent symptoms associated with SARS-CoV-2.

The 30-day, 60-day, and 90-day questionnaires (eTable 1 in Supplement 1) assessed baseline functional activity level and perceived symptoms in the 4 weeks prior to each survey. Each survey asked whether the patient felt that their health was back to normal. Baseline functional activity and maximal exertion level before COVID-19 were assessed by asking whether the patient could complete vigorous activities (eg, running), moderate activities (eg, moving a table), climb 1 flight of stairs, walk 1 block, lift or carry groceries, and bathe or dress independently.^26^ Patients were asked about 9 symptom clusters during the previous 4 weeks: fever, chills or night sweats; loss of smell or taste; fatigue; shortness of breath; chest pain; numbness or tingling; nausea, vomiting or diarrhea; muscle aches; and rash. Perceived cognitive deficits were evaluated with 3 questions modified from the Perceived Deficits Questionnaire, Fifth Edition^27^ that asked patients whether they had trouble getting things organized, whether they had trouble concentrating on activities like watching TV or reading a book, and whether they forgot what they talked about during a phone conversation during the previous 4 weeks. Likert scale response options included never, rarely, sometimes, often, and almost always. The Perceived Deficits Questionnaire, Fifth Edition has been studied in patients with multiple sclerosis, whiplash, and soft-tissue work injuries and has been found to be not associated with objective cognitive impairment, but instead with anxiety, depression, and self-efficacy.^28,29^

Demographic characteristics (age, sex, race, and ethnicity) were obtained from the electronic health record, as were a history of diabetes, organ transplant, body mass index (BMI; calculated as weight in kilograms divided by height in meters squared), Elixhauser Comorbidity Index,^30^ and facility of care for the initial SARS-CoV-2 infection (ie, inpatient facility or outpatient facility). Race and ethnicity (African American or Black, Asian, or White races, Hispanic or Latinx ethnicity, other race and/or ethnicity, or unknown) information were analyzed in this study because race and ethnicity have been associated with outcomes of SARS-CoV-2. Using the *International Statistical Classification of Diseases and Related Health Problems, Tenth Revision (ICD-10),* history of depression (*ICD-10* code F32), anxiety (*ICD-10* code F41), and cognitive difficulties (*ICD-10* codes F1, F2, and F3 for dementia; code R41 for cognitive decline; and code G31 for cognitive impairment) were obtained from encounter-associated codes in the electronic health record. Insurance was collapsed into commercial, Medicare, Medicaid, and none or other. A Social Vulnerability Index (SVI)^31^ was calculated and split into quartiles. Patients were characterized as having PCC if they noted persistent SARS-CoV-2 symptoms among the 9 symptom clusters noted above (none of which were cognitive or affective symptoms) on the 90-day survey or the 60-day survey if the 90-day survey was incomplete.

### Statistical Analysis

The 3 perceived cognitive deficit items from the questionnaire were scored from 0 to 4, and a mean of the 3 items was computed (α = .90). For the cohort of patients who completed the perceived cognitive deficits questions at the 30-day survey, we reported demographic and clinical characteristics, baseline functional activity status, history of depression, anxiety, or cognitive difficulties, and perceived cognitive deficits. We compared characteristics of patients reporting any perceived cognitive deficits vs no perceived cognitive deficits on the 30-day survey using *t* tests and χ^2^ tests.

To identify factors independently associated with perceived cognitive deficits on the 30-day survey, we trichotomized the perceived cognitive deficit score (0, >0 to 1.5, and >1.5 to 4) and performed ordinal logistic regression using this dependent variable. The proportional odds assumption was not violated by the χ^2^ score test. Independent variables included age in years (18–39, 40–59, and 60 or above), sex, race or ethnicity, health insurance, baseline functional activity status, clinical characteristics (diabetes, organ transplant, and BMI), SVI (in quartiles), inpatient vs outpatient care facility, and history of depression, anxiety, or cognitive difficulties. Multiple imputation was used for missing BMI (4 values), organ transplant (3 values), and Elixhauser Comorbidity Index score (81 values) after confirming that the data were missing at random. We also performed logistic regression analysis on the complete cases and observed no difference in the estimates (ie, the direction) and statistical significance (eTable 2 in Supplement 1).

We evaluated the association between patient-reported perceived cognitive deficits on the 30-day survey with their reports of PCC on later surveys. We compared characteristics of patients who reported PCC symptoms and those who did not report PCC symptoms using χ^2^ and *t* tests. A multivariable logistic regression model evaluated factors associated with report of PCC on 20 imputed data sets. The final odds ratio (OR) estimates were obtained by pooling the parameter estimates and associated covariance matrices for each imputation set. The prespecified factors included in the model^24^ included the aforementioned variables plus the trichotomized perceived cognitive deficits score. A 2-sided *P* < .05 was considered statistically significant. Analyses were performed using SAS statistical software version 9.4 (SAS Institute) from March 2022 to February 2023. To evaluate the longitudinal association of perceived cognitive deficits with PCC, we plotted the proportion of patients reporting any level of deficit for each cognitive deficit item, stratified by whether the patient reported PCC symptoms.

## Results

### Participant Characteristics

Of 1296 patients enrolled in the program from April 2020 to February 2021, 1038 patients completed any follow-up survey, and 766 patients (59% of the full cohort) completed the perceived cognitive deficits items on the survey approximately 30 days after hospital discharge or outpatient diagnosis. A total of 740 patients completed the 60-day survey, and 496 patients completed the 90-day survey. Of the 766 patients who completed the 30-day survey (mean [SD] age, 60.0 [16.7] years; 399 men [52.1%]; 317 Hispanic/Latinx patients [41.4%]; mean [SD] BMI, 30.0 [7.4]; median [IQR] SVI, 0.46 [0.20–0.76]), 293 patients (38.3%) had diabetes, and 90 patients (11.7%) had received an organ transplant. A total of 325 patients (42.4%) had commercial insurance. At baseline, 180 patients (23.5%) reported being able to complete vigorous activities and 368 patients (48.0%) reported being able to complete moderate activities. On the basis of encounter data, 109 patients (14.2%) had a history of cognitive difficulties, 153 patients (20.0%) had a diagnosis of depression, and 213 patients (27.8%) had a diagnosis of anxiety (Table 1).

### Perceived Cognitive Deficits Among Patients With SARS-CoV-2

During the 4 weeks following SARS-CoV-2 diagnosis, largely during the acute phase of illness, 490 of 766 patients (63.9%) reported no perceived cognitive deficits on the 3 cognitive survey items. A total of 231 patients (30.2%) reported trouble getting things organized, 220 patients (28.7%) reported trouble concentrating on activities like watching TV or reading a book, and 198 patients (25.8%) reported having forgotten what they had talked about during a telephone conversation. Of the 276 patients (36.1%) who perceived a cognitive deficit, 63 patients (22.8%) responded yes to only 1 item, 53 patients (19.2%) responded yes to 2 items, and 160 patients (58.0%) responded yes all 3 items. Overall, the mean (SD) perceived cognitive deficit score on the 30-day survey was 0.51 (0.82; median [IQR], 0.00 [0.00–1.00]); 164 patients (21.4%) had a mean score between 0.1 and 1.5, and 112 patients (14.6%) had a mean score above 1.5. During this period, the most common symptoms of acute COVID-19 were fatigue (432 patients [62%]), shortness of breath (475 patients [56%]), and muscle aches (345 patients [45%]) (eTable 2 in Supplement 1).

In bivariable analyses, no demographic or clinical factors other than diagnosis of depressive disorder, anxiety disorder, or cognitive difficulties and 1 aspect of physical function (moderate activities)were associated with patient report of a perceived cognitive deficit (Table 1). The logistic model demonstrates that a history of a cognitive difficulties (adjusted OR [aOR], 1.46; 95% CI, 1.16–1.83) and history of depressive disorder (aOR, 1.51; 95% CI, 1.23–1.86) were associated with a patient reporting cognitive deficits during the first 4 weeks after SARS-CoV-2. Women were more likely than men (aOR, 1.19; 95% CI, 1.01–1.40) and patients aged 40 to 59 years were more likely than younger patients (aOR, 1.35; 95% CI, 1.06–1.72) to report perceived cognitive deficits (Table 2).

### Association of Perceived Cognitive Deficits With PCC

Patients reporting cognitive deficits in the first 4 weeks after SARS-CoV-2 were more likely to report PCC symptoms at 60 to 90 days than those without perceived cognitive deficits (118 patients [42.8%] vs 105 patients [21.4%]; χ_1_^2^, 38.9; *P* < .001). Of the 223 patients with SARS-CoV-2 who reported PCC symptoms at 60 to 90 days, 118 patients (52.9%) reported a perceived cognitive deficit on the 30-day survey, whereas of the 543 patients with SARS-CoV-2 who did not report PCC symptoms at 60 to 90 days, 158 patients (29.1%) reported perceived cognitive deficits on the 30-day survey. Among patients reporting any perceived cognitive deficit, 64 of 164 patients (39.0%) with a mean perceived cognitive deficit score below 1.5 reported PCC symptoms as did 54 of 112 patients (48.2%) with a mean perceived cognitive deficit score above 1.5. The association of patient demographic and clinical characteristics and whether patients reported PCC symptoms is displayed in Table 3. History of depressive disorder, cognitive difficulties, and diabetes were associated with reporting of PCC symptoms, and organ transplant was negatively associated with PCC symptoms. Perceived cognitive score was most associated with reporting PCC symptoms at the 60-day to 90-day survey (Table 3).

In the logistic model of PCC, perceived cognitive deficits were associated with reporting of PCC symptoms, and there was a dose-response association between severity of perceived cognitive deficits and likelihood of reporting PCC symptoms. Compared with patients reporting no perceived cognitive deficits, patients reporting a perceived cognitive deficit with a mean score of 1.5 or less were 2.42 times (95% CI, 1.52–3.60) more likely to report PCC symptoms and those with a score above 1.5 were 2.97 times (95% CI, 1.86 – 4.75) more likely to report PCC symptoms. Patients with diabetes (OR, 1.60; 95% CI, 1.11–2.30) were more likely to report PCC symptoms, whereas those with organ transplants (OR, 0.49; 95% CI, 0.26–0.92) or Medicaid insurance (OR, 0.54; 95% CI, 0.31–0.95) were less likely to report PCC symptoms (Table4). We also performed logistic regression analysis on the complete cases and observed no difference in the estimates (ie, the direction) and statistical significance (eTable 3 in Supplement 1).

The proportion of patients reporting cognitive deficits at the question level, (Figure) shows that for patients who subsequently did not report PCC symptoms, proportions of patients perceiving persistent cognitive deficits at the 60-day and 90-day surveys decreased. However, among patients reporting PCC symptoms, perception of cognitive deficits remained about the same over the 3-month study period.

## Discussion

In this cohort study, more than one-third of patients with SARS-CoV-2 perceived cognitive deficits on the 30-day survey after hospitalization or outpatient infection. Report of perceived cognitive deficit was associated with later reporting of PCC symptoms. To some degree, these findings may help us disentangle the complex construct that is PCC. Prior use of these survey items demonstrates that perceived cognitive deficit is not associated with objective deficient cognition; instead, they are associated with depression, anxiety, and lower perceived functional ability and control.^28,29^ Those findings are consistent with the findings in this cohort study showing that perceived cognitive deficits were associated with a history of anxiety disorder and depressive disorder, although we also found an association with prior cognitive difficulties. These findings suggest a substantial psychological component for long lasting SARS-CoV-2 symptoms for at least some patients.

These findings are also consistent with literature suggesting that PCC is a heterogeneous condition.^2^ Nearly one-half of the patients with PCC reported no perceived cognitive deficits. The temporal trends show that the prevalence of perceived cognitive deficits declines among patients who did not report PCC symptoms while perceived cognitive deficits remained stable among those who reported PCC symptoms at 60 to 90 days. Furthermore, as seen in the model estimating PCC, perceived severity of the reported cognitive deficits is associated with the probability of later reporting PCC symptoms.

Many reports show that cognitive impairment and memory difficulty are common among patients with acute SARS-CoV-2,^17,32^ and among those with PCC.^2,8,11–14^ However, many studies use convenience samples or have no longitudinal data. A 2022 report^23^ of a large sample of nurses (albeit with little gender or race heterogeneity) demonstrated that pre-SARS-CoV-2 distress, both general distress and COVID-19–related distress, was associated with greater likelihood of COVID-19 symptoms persisting for 4 or more weeks. Our findings come from a single health system continuity sample, and we are able to adjust the diverse cohort for demographic and clinical characteristics.^24^

The finding that more than one-half of patients with PCC perceived cognitive deficits early during the condition is provocative, yet these data likely create more questions than they answer. Do the reported cognitive deficits influence the content or quality of responses to later surveys? Are the early reported cognitive deficits related to the SARS-CoV-2 infection consistent with imaging changes that have been found ?^33,34^ If so, why are these symptoms related to a history of depressive disorder, anxiety disorder, and cognitive difficulties? These data suggest that the constructs of affect and control play a substantial role in the development of PCC for at least some patients. From a clinical perspective, these data might suggest that early evaluation of perceived cognitive deficits might help in identification of patients with acute COVID-19 who should receive more intensive monitoring for persistence of symptoms and perhaps for a focus on intervention.

### Limitations

This study has several limitations, including a lack of objective measures of cognition because these clinical surveys aimed to identify patients at risk of clinical deterioration. The principal survey items that elicit subjective responses about perceived cognitive deficits have not been shown to correlate with objective cognitive deficits. Measures of pre-SARS-CoV-2 cognition, depression, and anxiety were obtained from clinical encounter data, which is known to miss these diagnoses.^35,36^ In addition, the definition of PCC may be biased because it is a subjective rating of a limited number of symptoms. Furthermore, referral bias may exist among the outpatient cohort, because physicians may have referred patients deemed clinically high risk to the program, which may affect generalizability of the outpatient cohort. The patient cohort studied was derived from an academic medical center, indicating that it may not be generalizable to other groups of patients with COVID-19.

## Conclusions

In a longitudinal cohort study of patients with SARS-CoV-2 in 1 health care system, we found an association between perceived cognitive deficits early in the disease and PCC, which suggests direction for exploration of the underpinnings of PCC.

## Supplementary Material

Supplemental material 1

Supplemental material 2

## Figures and Tables

**Figure.**
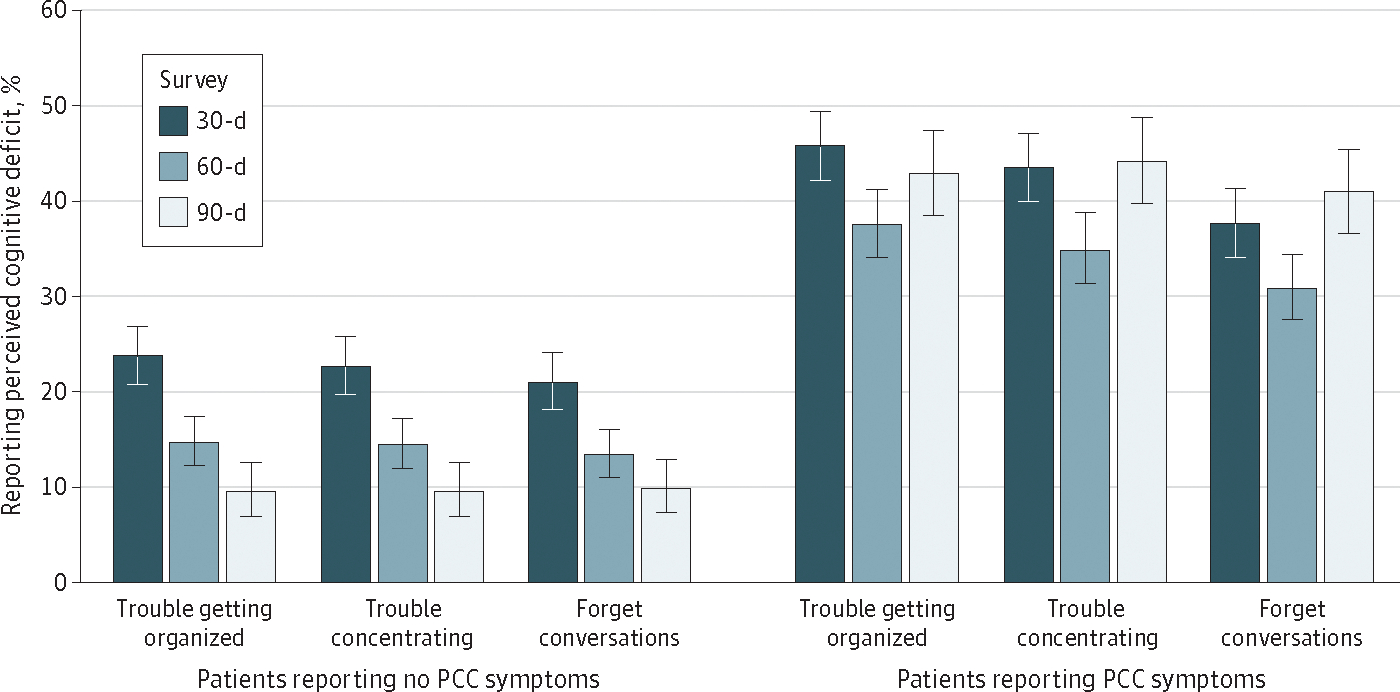
Comparison of Patients With and Without Symptoms of Post–COVID-19 Condition (PCC) and Perceived Cognitive Deficits Over Time Figure shows patient responses to perceived cognitive deficit items from the 30-day, 60-day, and 90-day surveys, comparing patients who did or did not report PCC symptoms at the 60-day or 90-day survey. Error bars denote 95% CIs.

**Table 1.** Demographic and Clinical Characteristics of Patients

Characteristic	Patients, No. (%)			OR (95% CI)
	Total (N = 766)	Reporting a perceived cognitive deficit (n = 276)^a^	Reporting no perceived cognitive deficit (n = 490)	
Age, mean, (SD), y	60.0 (16.7)	59.6 (17.1)	60.2 (16.5)	NA
Age range, y				
18–39	105 (13.7)	36 (13.0)	69 (14.1)	1 [Reference]
40–59	257 (33.6)	104 (37.7)	153 (31.2)	1.30 (0.81–2.09)
≥60	404 (52.7)	136 (49.3)	268 (54.7)	0.97 (0.62–1.53)
Sex				
Female	367 (47.9)	144 (52.2)	223 (45.5)	1.31 (0.97–1.76)
Male	399 (52.1)	132 (47.8)	267 (54.5)	1 [Reference]
Race and ethnicity				
African American or Black	58 (7.8)	29 (10.5)	29 (5.9)	1.56 (0.88–2.78)
Asian	65 (8.5)	24 (8.7)	41 (8.4)	0.91 (0.52–1.61)
Hispanic or Latinx	317 (41.4)	99 (35.9)	218 (44.7)	0.71 (0.50–1.01)
White	233 (30.4)	91 (33)	142 (29.4)	1 [Reference]
Other race and ethnicity or unknown^b^	93 (12.1)	33 (12)	60 (11.6)	0.86 (0.52–1.42)
Comorbidities^c^				
Diabetes	293 (38.3)	101 (36.6)	192 (39.2)	0.90 (0.66–1.22)
Organ transplant	90 (11.7)	25 (9.1)	65 (13.3)	0.65 (0.40–1.06)
Body mass index, mean (SD)^d^	30.0 (7.4)	30.5 (7.6)	29.7 (7.2)	1.01 (0.99–1.04)
History of neuropsychiatric condition^c^				
Depressive disorder	153 (20.0)	81 (29.3)	72 (14.7)	2.41 (1.68–3.46)
Anxiety disorder	213 (27.8)	95 (34.4)	118 (24.1)	1.66 (1.20–2.29)
Cognitive difficulties (dementia, cognitive decline, or cognitive impairment)	109 (14.2)	57 (20.7)	52 (10.6)	2.19 (1.46–3.30)
Health care facility				
Outpatient	187 (24.4)	63 (22.8)	124 (25.3)	1 [Reference]
Inpatient	579 (75.6)	213 (77.2)	366 (74.7)	1.15 (0.81–1.62)
Social Vulnerability Index, percentile				
0–25	226 (29.5)	86 (31.2)	140 (28.6)	1 [Reference]
25.1–50	163 (21.3)	61 (22.1)	102 (20.8)	0.87 (0.64–1.48)
50.1–75	148 (19.3)	48 (17.4)	100 (20.4)	0.78 (0.51–1.21)
75.1–100	193 (25.2)	66 (23.9)	127 (25.9)	0.85 (0.57–1.26)
Missing	36 (4.7)	15 (5.4)	21 (4.3)	1.16 (0.57–2.38)
Elixhauser ComorbidityIndex score, mean (SD)^d^	10.9 (12.5)	11 (13.5)	10.8 (12)	NA
Health insurance,				
Commercial	325 (42.4)	123 (44.6)	202 (41.2)	1 [Reference]
Medicare	290 (37.9)	98 (35.5)	192 (39.2)	0.84 (0.60–1.17)
Medicaid	126 (16.5)	45 (16.3)	81 (16.5)	0.91 (0.60–1.40)
Other or none	25 (3.3)	10 (3.6)	15 (3.1)	1.10 (0.48–2.51)
Baseline functional activity status				
Vigorous	180 (23.5)	77 (27.9)	103 (21.0)	1 [Reference]
Moderate	368 (48.0)	107 (38.8)	261 (53.3)	1.82 (1.26–2.64)
Able to climb 1 flight stairs or walk 1 block	53 (8.2)	22 (8.0)	31 (6.3)	1.05 (0.57–1.96)
Able to carry groceries, bathe, or dress	151 (19.7)	62 (22.5)	89 (18.2)	1.07 (0.69–1.66)
Missing	14 (1.8)	8 (2.9)	6 (1.2)	NA

Abbreviations: NA, not applicable; OR, odds ratio.

a Perceived cognitive deficits were reported at the 30-day interview conducted approximately 4 weeks after hospital discharge or ambulatory infection.

b Other was defined as patients who chose multiple races or ethnicities or who chose other race or ethnicity.

c Reference for diabetes is no diabetes, for organ transplant is no organ transplant, and for neuropsychiatric conditions is no condition.

d Unit for body mass index and Elixhauser Comorbidity Index is 1 unit.

**Table 2.** Factors Associated With Perceived Cognitive Deficits at 30-Day Interview After Initial SARS-CoV-2 Infection^a^

Factor	Adjusted OR (95% CI)
Age range, y	
18–39	1 [Reference]
40–59	1.35 (1.06–1.72)
≥60	0.88 (0.67–1.16)
Sex	
Female	1.19 (1.01–1.40)
Male	1 [Reference]
Race and ethnicity	
African American or Black	1.55 (1.00–2.40)
Asian	0.97 (0.62–1.51)
Hispanic or Latino	0.78 (0.52–1.18)
White	1 [Reference]
Other race and ethnicity or unknown^b^	0.97 (0.66–1.42)
Comorbidities^c^	
Diabetes	0.88 (0.63–1.24)
Organ transplant	0.74 (0.43–1.28)
Body mass index^d^	1.01 (0.99–1.03)
Health insurance	
Commercial	1 [Reference]
Medicare	0.79 (0.55–1.12)
Medicaid	1.05 (0.72–1.54)
Other or none	1.20 (0.64–2.28)
Social Vulnerability Index, percentile	
0–25	1 [Reference]
25.1–50	0.85 (0.63–1.16)
50.1–75	0.82 (0.59–1.13)
75.1–100	1.06 (0.75–1.48)
Missing	1.21 (0.70–2.11)
Elixhauser ComorbidityIndex score^d^	1.00 (0.98–1.01)
Health care facility	
Inpatient	1.40 (0.96–2.05)
Outpatient	1 [Reference]
Baseline functional activity status	
Vigorous	1 [Reference]
Moderate	0.49 (0.21–1.15)
Able to climb 1 flight stairs or walk 1 block	1.22 (0.85–1.75)
Able to carry groceries, bathe, or dress	0.58 (0.39–0.88)
Missing	1.74 (0.74–4.06)
History of neuropsychiatric condition^c^	
Cognitive difficulties (dementia, cognitive decline, or cognitive impairment)	1.46 (1.16–1.83)
Depressive disorder	1.51 (1.23–1.86)
Anxiety disorder	1.08 (0.90–1.31)

Abbreviation: OR, odds ratio.

a Perceived cognitive deficits were reported at the 30-day interview conducted approximately 4 weeks after hospital discharge or ambulatory infection. Ordinal logistic regression was performed for 766 patients.

b Other was defined as patients who chose multiple races or ethnicities or who chose other race or ethnicity.

c Reference for diabetes is no diabetes, for organ transplant is no organ transplant, and for neuropsychiatric conditions is no condition.

d Unit for body mass index and Elixhauser Comorbidity Index is 1 unit.

**Table 3.** Association of Patient Demographics, Clinical Characteristics, and Perceived Cognitive Deficits With Report of PCC Symptoms at 60–90 Day Survey

Characteristic	Patients, No. (%) (N = 766)		OR (95% CI)
	No PCC symptoms (n = 543)	PCC symptoms (n = 223)	
Age range, y			
18–39	77 (14.2)	28 (12.6)	1 [Reference]
40–59	180 (33.1)	77 (34.5)	1.18 (0.71–1.96)
≥60	286 (52.7)	118 (52.9)	1.14 (0.70–1.84)
Sex			
Female	255 (47.0)	112 (50.2)	1.14 (0.83–1.56)
Male	288 (53.0)	111 (49.8)	1 [Reference]
Race and ethnicity			
African American or Black	40 (7.4)	18 (8.1)	1.04 (0.56–1.94)
Asian	46 (8.5)	19 (8.5)	0.95 (0.52–1.74)
Hispanic or Latino	226 (41.6)	92 (41.3)	0.94 (0.65–1.36)
White	164 (30.2)	71 (31.8)	1 [Reference]
Other race and ethnicity or unknown^a^	67 (12.3)	23 (10.3)	0.79 (0.46–1.37)
Comorbidities^b^			
Diabetes	194 (35.7)	99 (44.4)	1.44 (1.05–1.97)
Organ transplant	73 (13.4)	17 (7.6)	0.53 (0.31–0.92)
Body mass index			
<30	327 (60.2)	122 (54.7)	1 [Reference]
≥30	216 (39.8)	101 (45.3)	1.25 (0.92–1.72)
History of neuropsychiatric condition^b^			
Depressive disorder	94 (17.3)	59 (26.5)	1.72 (1.19–2.49)
Anxiety disorder	141 (26.0)	72 (32.3)	1.36 (0.97–1.91)
Cognitive difficulties (dementia, cognitive decline, or cognitive impairment)	65 (12.0)	44 (19.7)	1.81 (1.19–2.75)
Health care facility,			
Outpatient	140 (25.8)	47 (21.1)	1 [Reference]
Inpatient	403 (74.2)	176 (78.9)	1.30 (0.89–1.89)
Social Vulnerability Index, percentile			
0–25	159 (29.3)	67 (30.0)	1 [Reference]
25.1–50	118 (21.7)	45 (20.2)	0.91 (0.58–1.41)
50.1–75	104 (19.2)	44 (19.7)	1.00 (0.64–1.58)
75.1–100	138 (25.4)	55 (24.7)	0.95 (0.62–1.44)
Missing	24 (4.4)	12 (5.4)	1.19 (0.56–2.51)
Elixhauser ComorbidityIndex score,			
<10	262 (48.3)	105 (47.1)	1 [Reference]
≥10	224 (41.3)	94 (42.2)	1.05 (0.75–1.46)
Health insurance			
Commercial	224 (41.3)	101 (45.3)	1 [Reference]
Medicare	204 (37.6)	86 (38.6)	0.94 (0.66–1.32)
Medicaid	98 (18.0)	28 (12.6)	0.63 (0.39–1.03)
Other or none	17 (3.1)	8 (3.6)	1.04 (0.44–2.50)
Baseline functional activity status			
Vigorous	125 (23.0)	55 (24.7)	1 [Reference]
Moderate	266 (49.0)	102 (45.7)	0.87 (0.59–1.29)
Able to climb 1 flight stairs or walk 1 block	117 (21.5)	50 (22.4)	0.97 (0.61–1.54)
Able to carry groceries, bathe, or dress	25 (4.6)	12 (5.4)	1.09 (0.51–2.33)
Missing	10 (1.8)	4 (1.8)	0.91 (0.27–3.03)
Perceived cognitive deficit score^c^			
0	385 (70.9)	105 (47.1)	1 [Reference]
>0 to 1.5	100 (18.4)	64 (28.7)	2.35 (1.60–3.43)
>1.5 to 4	58 (10.7)	54 (24.2)	3.41 (2.22–5.24)

Abbreviations: OR, odds ratio; PCC, post-COVID-19 condition.

a Other was defined as patients who chose multiple races or ethnicities or who chose other race or ethnicity.

b Reference for diabetes is no diabetes, for organ transplant is no organ transplant, and for neuropsychiatric conditions is no condition.

c Perceived cognitive deficits reported at the 30-day survey conducted approximately 4 weeks after hospital discharge or ambulatory infection.

**Table 4.** Factors Associated With Reporting of PCC Symptoms After Ordinal Logistic Regression

Factor	OR (95% CI)
Age range, y	
18–39	1 [Reference]
40–59	0.98 (0.56–1.72)
≥60	1.01 (0.55–1.85)
Sex	
Female	1.05 (0.73–1.50)
Male	1 [Reference]
Race and ethnicity	
African American or Black	0.89 (0.45–1.78)
Asian	0.96 (0.50–1.86)
Hispanic or Latinx	1.08 (0.68–1.74)
White	1 [Reference]
Other race and ethnicity or unknown^a^	0.81 (0.45–1.47)
Comorbidities^b^	
Diabetes	1.60 (1.11–2.30)
Organ transplant	0.49 (0.26–0.92)
Body mass index^c^	1.01 (0.98–1.03)
Health insurance	
Commercial	1 [Reference]
Medicare	0.92 (0.05–1.43)
Medicaid	0.54 (0.31–0.95)
Other or none	0.99 (0.39–2.51)
Social Vulnerability Index, percentile	
0–25	1 [Reference]
25.1–50	0.90 (0.55–1.47)
50.1–75	1.04 (0.62–1.75)
75.1–100	1.13 (0.65–1.98)
Missing	1.17 (0.52–2.65)
Elixhauser Comorbidity Index score^c^	0.99 (0.98–1.01)
Health care facility	
Inpatient	1.45 (0.95–2.22)
Outpatient	1 [Reference]
Baseline functionalactivity status	
Vigorous	1 [Reference]
Moderate	0.98 (0.63–1.52)
Able to climb 1 flight stairs or walk 1 block	0.81 (0.47–1.40)
Able to carry groceries, bathe. or dress	1.00 (0.41–2.43)
Missing	0.52 (0.13–2.04)
History of neuropsychiatric condition^b^	
Cognitive difficulty (dementia, cognitive decline, or cognitive impairment)	1.48 (0.88–2.46)
Depressive disorder	1.12 (0.70–1.79)
Anxiety disorder	1.25 (0.82–1.88)
Perceived Cognitive Deficits score	
0	1 [Reference]
>0 to 1.5	2.42 (1.62–3.60)
>1.5 to 4	2.97 (1.86–4.75)

Abbreviations: OR, odds ratio; PCC, post-COVID-19 condition.

a Other was defined as patients who chose multiple races or ethnicities or who chose other race or ethnicity.

b Reference for diabetes is no diabetes, for organ transplant is no organ transplant and for neuropsychiatric conditions is no condition.

c Unit for body mass index and Elixhauser Comorbidity Index is 1 unit.

## Data Availability

See Supplement 2.

## Key Points

### Question

Among hospitalized and ambulatory patients with SARS-CoV-2 infection, how many perceived cognitive deficits early in the course of their infection and were these perceived deficits associated with post–COVID-19 condition (PCC)?

### Findings

In this cohort study of 766 patients with SARS-CoV-2 infection, 276 patients (36.1%) perceived cognitive deficits within 4 weeks of hospital discharge or outpatient infection, and patients with perceived cognitive deficits at 30 days were twice as likely as patients without perceived deficits to report symptoms of PCC at 60 to 90 days. Severity and persistence of perceived cognitive deficits were associated with symptoms of PCC.

### Meaning

These findings of perceived cognitive deficits early during the course of SARS-CoV-2 infection suggest that there is an affective component to PCC in some patients.
