# Supplementary material for: Perceived Cognitive Deficits in Patients With Symptomatic SARS-CoV-2 and Their Association With Post–COVID-19 Condition
Source: JAMA Netw Open. 2023 May 5;6(5):e2311974. doi: 10.1001/jamanetworkopen.2023.11974 (PMC10715897; doi:10.1001/jamanetworkopen.2023.11974)
Supplement: Supplement 1. — eTable 1. Clinical and Functional Survey eTable 2. Symptoms During Acute COVID-19 and at 30-Day Interview eTable 3. Complete Case Analysis: Factors Associated With Development of Long COVID [file jamanetwopen-e2311974-s001.pdf]

Supplemental Online Content

Liu TC, Yoo SM, Sim MS, Motwani Y, Viswanathan N, Wenger NS. Perceived cognitive deficits in patients with symptomatic SARS-CoV-2 and their association with post–COVID-19 condition. *JAMA Netw Open*. 2023;6(5):e2311974. doi:10.1001/jamanetworkopen.2023.11974

eTable 1. Clinical and Functional Survey
eTable 2. Symptoms During Acute COVID-19 and at 30-Day Interview
eTable 3. Complete Case Analysis: Factors Associated With Development of Long COVID

This supplemental material has been provided by the authors to give readers additional information about their work.

© 2023 Liu TC et al. *JAMA Network Open.*

eTable 1. Clinical and Functional Survey

Information obtained by trained clinical staff (RNs) at 30 days, 60 days, and 90 days after acute illness or hospitalization.

**1**	Do you feel your health is back to normal? (If YES, skip next question 3)	☐ Yes ☐ No
**2**	Compare to 4 weeks ago, how would you rate your health now? (1=way worse than prior baseline; 10=back to normal)	
**3**	During the past 4 weeks, has your physical health interfered with your normal social activities with family, friends, neighbors, or groups?	☐ Yes ☐ No<br>Explain:
**4**	During the past 4 weeks, have you had any problems with your work or other regular daily activities as a result of your physical health?	☐ Yes ☐ No<br>Explain:
**5**	What is the maximal exertion you could do BEFORE COVID-19 diagnosis?<br>(Choose the highest level of activity you were able to do)	☐ Vigorous activities, such as running, lifting heavy objects, participating in strenuous sports<br>☐ Moderate activities, such as moving a table, pushing a vacuum cleaner, bowling, or playing golf<br>☐ Climbing one flight of stairs<br>☐ Walking one block<br>☐ Lifting or carrying groceries<br>☐ Bathing or dressing yourself
**6**	Does your health now limit you in these activities? If so, how much?	
	Vigorous activities, such as running, lifting heavy objects, participating in strenuous sports	☐ Yes, limited a lot<br>☐ Yes, limited a little<br>☐ No, not limited at all
	Moderate activities, such as moving a table, pushing a vacuum cleaner, bowling, or playing golf	☐ Yes, limited a lot<br>☐ Yes, limited a little<br>☐ No, not limited at all
	Climbing one flight of stairs	☐ Yes, limited a lot<br>☐ Yes, limited a little<br>☐ No, not limited at all
	Walking one block	☐ Yes, limited a lot<br>☐ Yes, limited a little<br>☐ No, not limited at all
	Lifting or carrying groceries	☐ Yes, limited a lot<br>☐ Yes, limited a little<br>☐ No, not limited at all
	Bathing or dressing yourself	☐ Yes, limited a lot<br>☐ Yes, limited a little

© 2023 Liu TC et al. *JAMA Network Open.*

		☐ No, not limited at all
**7**	After the COVID-19 diagnosis, how often did you	
	Have trouble getting things organized during the last 4 weeks?	☐ Never<br>☐ Rarely<br>☐ Sometimes<br>☐ Often<br>☐ Almost always
	Have trouble concentrating on things like watching television program or reading a book during the last 4 weeks?	☐ Never<br>☐ Rarely<br>☐ Sometimes<br>☐ Often<br>☐ Almost always
	Forget what you talked about after a telephone conversation?	☐ Never<br>☐ Rarely<br>☐ Sometimes<br>☐ Often<br>☐ Almost always
**8**	Have you had any of the following symptoms over last 4 weeks? Have the symptoms resolved?	☐ Fever, chills or night sweats – ☐ Resolved<br>☐ Loss of smell or taste -- ☐ Resolved<br>☐ Fatigue -- ☐ Resolved<br>☐ Shortness of breath -- ☐ Resolved<br>☐ Chest pain -- ☐ Resolved<br>☐ Numbness or tingling -- ☐ Resolved<br>☐ Nausea, vomiting, or diarrhea -- ☐ Resolved<br>☐ Muscle aches -- ☐ Resolved<br>☐ Rashes -- ☐ Resolved

© 2023 Liu TC et al. *JAMA Network Open.*

eTable 2. Symptoms During Acute COVID-19 and at 30-Day Interview

	Present during acute COVID	Present at 30-days after COVID
Fever, chills or night sweats	43%	0.8%
Loss of smell or taste	32%	6%
Fatigue	62%	20%
Shortness of breath	56%	11%
Chest pain	20%	3%
Numbness or tingling	12%	3%
Nausea, vomiting or diarrhea	30%	2%
Muscle aches	45%	5%
Rash	9%	0.5%

© 2023 Liu TC et al. *JAMA Network Open.*

eTable 3. Complete Case Analysis: Factors Associated With Development of Long COVID

	**Odds Ratio (95% Confidence interval)**
Age	
18-39	Ref
40-59	0.95 (0.51 - 1.78)
60+	0.97 (0.50 - 1.88)
Sex	
Female	1.03 (0.70 - 1.50)
Male	Ref
Race	
Black	0.93 (0.45 - 1.93)
Asian	0.87 (0.43 - 1.74)
Latino	1.06 (0.64 - 1.75)
Other/Unknown	0.70 (0.35 - 1.38)
White	Ref
Comorbidities	
Diabetes	1.74 (1.19 - 2.55)
Organ transplant	0.48 (0.25 - 0.91)
Body mass index	1.00 (0.98 - 1.03)
Insurance	
Commercial	Ref
Medicare	0.83 (0.53 - 1.31)
Medicaid	0.54 (0.30 - 0.98)
Other	0.83 (0.53 - 1.31)
Social Vulnerability Index	
0-25 percentile	Ref
25-50th percentile	0.81 (0.48 - 1.36)
50-75th percentile	0.86 (0.49 - 1.51)
>75th percentile	1.07 (0.59 - 1.93)
Missing	1.17 (0.47 - 2.88)
Elixhauser comorbidity score	1.00 (0.98 - 1.01)
Venue of care	
Inpatient	1.56 (0.99 - 2.46)
Outpatient	Ref
Baseline functional status	
Vigorous	Ref
Moderate	0.86 (0.53 - 1.40)
1 flight stairs or walk 1 block	0.64 (0.36 - 1.16)
Carry groceries/bathe/dress	0.97 (0.39 - 2.42)
Missing	0.47 (0.12 - 1.87)
History of neurocognitive condition	
Cognitive decline	1.48 (0.87 – 2.51)
Depressive disorder	1.14 (0.70 – 1.85)
Anxiety disorder	1.04 (0.67 – 1.61)
Perceived Cognitive Deficits score	
0	Ref
>0 to 1.5	2.51 (1.63 – 3.85)
>1.5 to 4	2.79 (1.69 – 4.59)

Ordinal logistic regression, n=679 complete cases
Reference for Diabetes is No diabetes, for Organ transplant is No organ transplant. Unit for Body mass index and Elixhauser comorbidity index is 1 unit.

© 2023 Liu TC et al. *JAMA Network Open.*
