# Supplementary material for: Perceived Cognitive Deficits in Patients With Symptomatic SARS-CoV-2 and Their Association With Post–COVID-19 Condition
Source: JAMA Netw Open. 2023 May 5;6(5):e2311974. doi: 10.1001/jamanetworkopen.2023.11974 (PMC10715897; doi:10.1001/jamanetworkopen.2023.11974)
Supplement: Supplement 2. — Data Sharing Statement [file jamanetwopen-e2311974-s002.pdf]

Data Sharing Statement

Liu. Perceived Cognitive Deficits in Patients With Symptomatic SARS-CoV-2 and Their Association With Post-COVID-19 Condition. *JAMA Netw Open*. Published May 05, 2023. doi:10.1001/jamanetworkopen.2023.11974

Data
Data available: No

Additional Information
Explanation for why data not available: Analysis is ongoing
